# Supplementary material for: Unraveling the synergistic effects of Cu-Ag tandem catalysts during electrochemical CO2 reduction using nanofocused X-ray probes
Source: Nat Commun. 2023 Nov 29;14:7833. doi: 10.1038/s41467-023-43693-2 (PMC10687089; doi:10.1038/s41467-023-43693-2)
Supplement: Supplementary file 1 — Supplementary Information [file 41467_2023_43693_MOESM1_ESM.pdf]

Supplementary Information

Unraveling the Synergistic Effects of Cu-Ag Tandem Catalysts during Electrochemical CO_2 Reduction using Nanofocused X-ray Probes

Marvin L. Frisch[1,†], Longfei Wu[1,2,†], Clément Atlan[3,4], Zhe Ren[5], Madeleine Han[3], Rémi Tucoulou[3], Liang Liang[1], Jiasheng Lu[1], An Guo[1], Hong Nhan Nong[1], Aleks Arinchtein[1], Michael Sprung[5], Julie Villanova[3], Marie-Ingrid Richard[3,4], and Peter Strasser[1,*]

[1]Department of Chemistry, Chemical Engineering Division, Technische Universitaet Berlin, Str. des 17. Juni 124, 10623 Berlin, Germany.

[2]Alexander von Humboldt Foundation, Jean-Paul-Str. 12, 53173 Bonn, Germany.

[3]ESRF, The European Synchrotron, 71 Avenue des Martyrs, Grenoble 38000, France.

[4]CEA Grenoble, IRIG/MEM/NRX, Université Grenoble Alpes, Grenoble 38054, France.

[5]Deutsches Elektronen-Synchrotron (DESY), Notkestr. 85, 22607 Hamburg, Germany.

[†]These authors contributed equally: Marvin L. Frisch, Longfei Wu.

[*]email: pstrasser@tu-berlin.de

This file contains Supplementary Figs. 1 - 15.

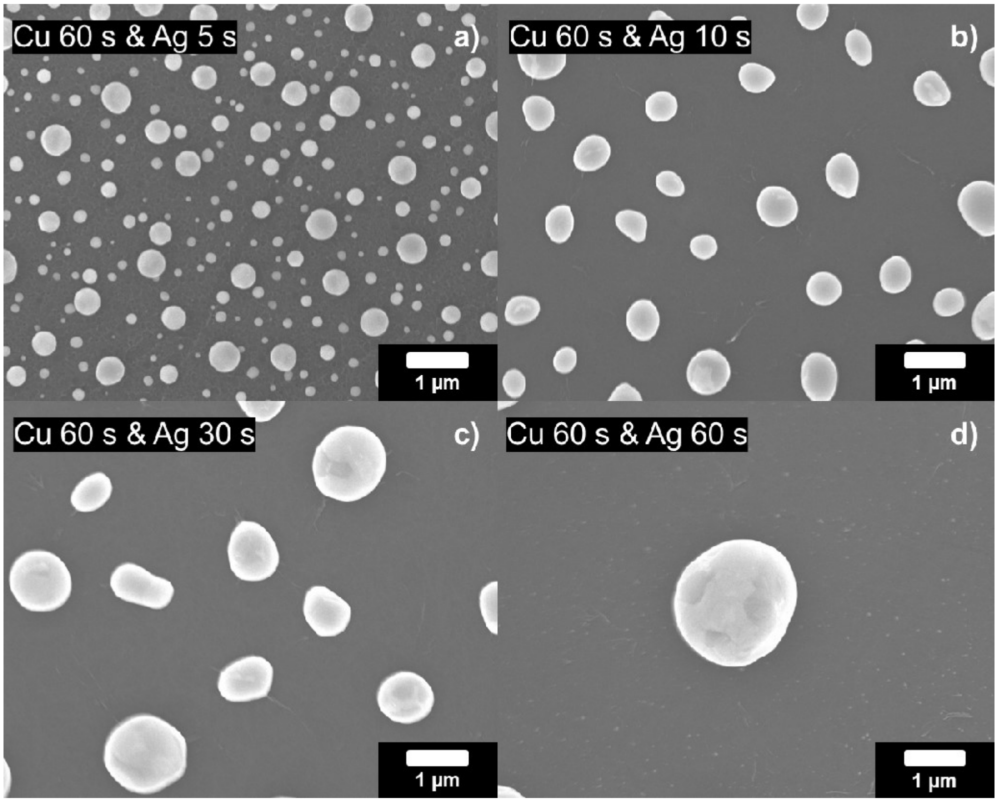

Supplementary Fig. 1 SEM images (a-d) of different Cu-Ag tandem NPs synthesized *via* sputter-coating and subsequent dewetting at 750 °C in H_2/Ar. By varying the deposition time for Ag between 5 and 60 s, the composition and the average particle size can be tuned. With increasing Ag content, the average particle size is found to increase after the dewetting step. For nanofocused X-ray experiments, average particle sizes between 100 and 500 nm are favorable. Additionally, well-dispersed particles featuring a similar morphology and narrow size distribution are highly desirable for reliable *in situ* investigations using nanofocused X-rays.

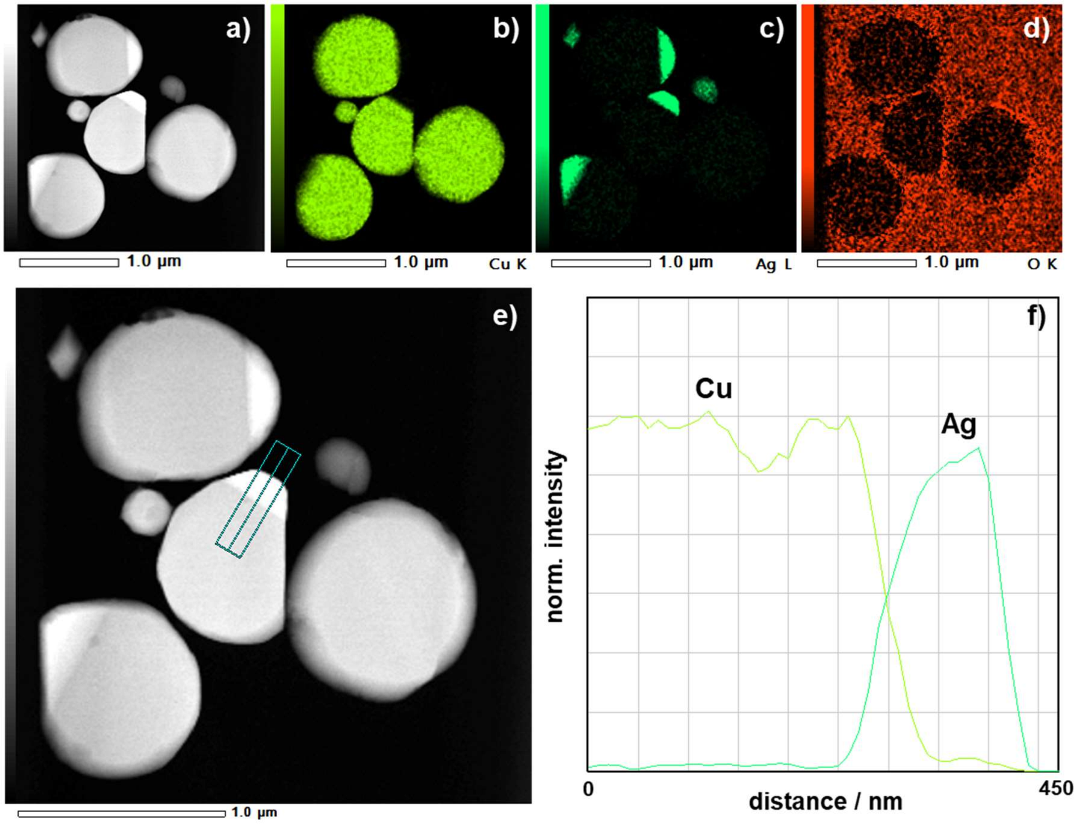

Supplementary Fig. 2 ADF-STEM/EDX mappings of the as-prepared bimetallic $Cu_{0.88}Ag_{0.12}$ tandem catalyst particles. A Janus-type structure with phase-segregated Cu and Ag domains can be observed (a-e). At the interface, both Cu and Ag species are present showing a gradient in composition, which is further highlighted by the evaluation of the representative EDX line-scan (e, f).

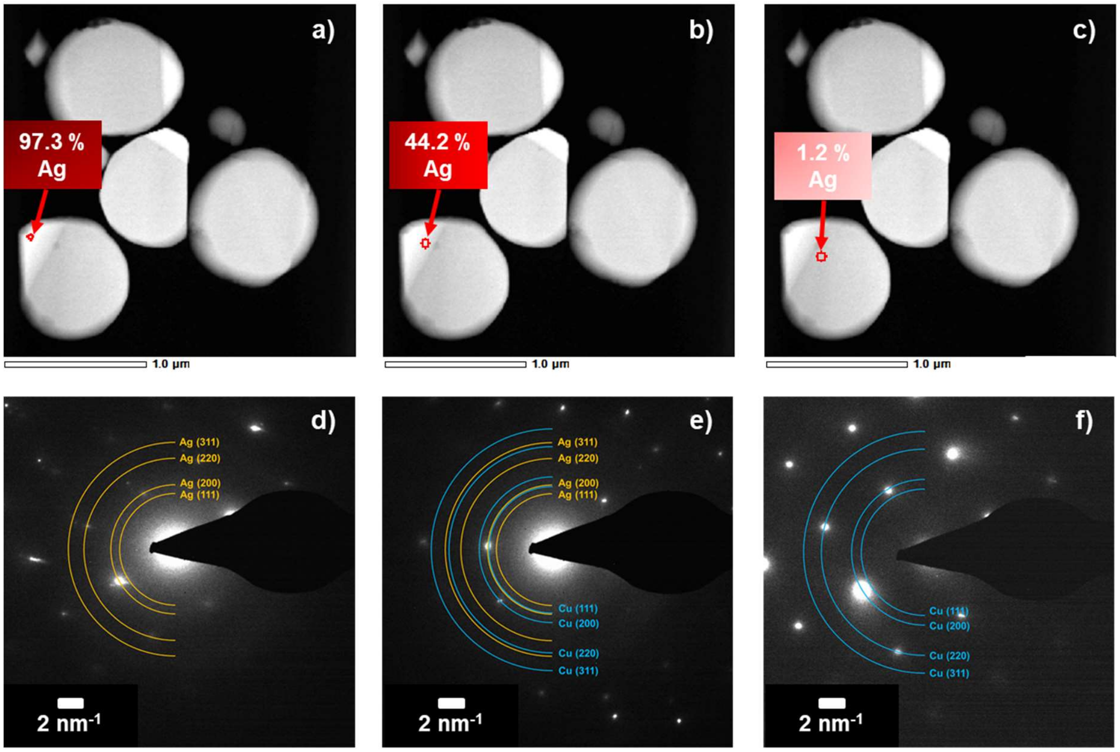

Supplementary Fig. 3 ADF-STEM/EDX evaluation (a-c) and SAED patterns (d-f) of the as-prepared bimetallic $Cu_{0.88}Ag_{0.12}$ tandem catalyst particles. Combination of EDX and SAED analyses indicate the presence of a small, Ag-rich alongside a large, Cu-rich domain of brighter and darker contrast (a-c), respectively. In (d-f), reference patterns for Ag (PDF no. 01-089-3722) and Cu (PDF no. 01-089-2838) are illustrated in orange and blue, respectively. At the interface (b, e), both metals are present in the as-prepared particles. Note that the streaking of the diffraction spots in (d) indicates the presence of Cu^0 alongside the predominant Ag^0 phase. This effect may also be related to the large NP size in the range of several hundred nanometers.

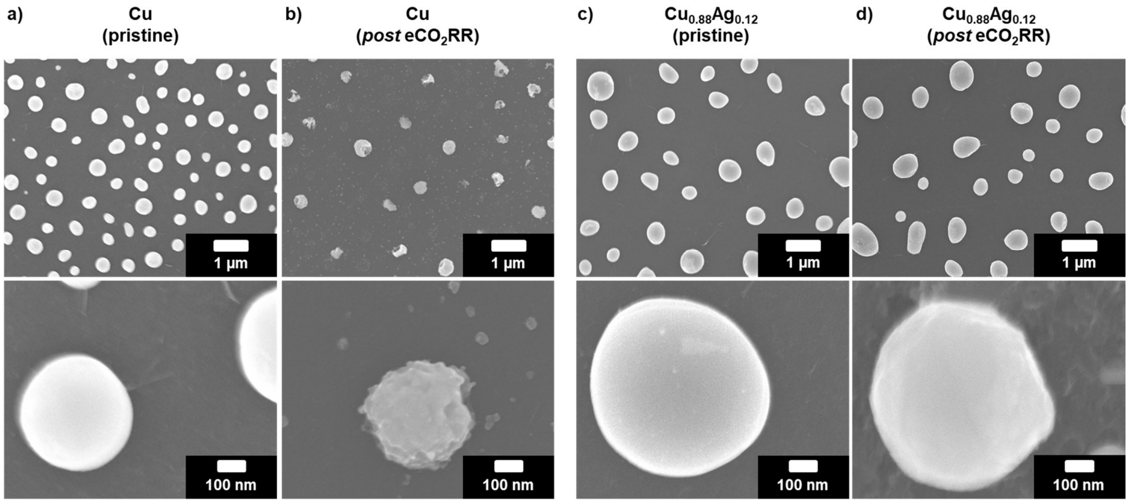

Supplementary Fig. 4 SEM images of pristine Cu as well as of $Cu_{0.88}Ag_{0.12}$ NPs prior to (a, c) and after (b, d) eCO_2RR. During the 6 h eCO_2RR test, bare Cu NPs undergo severe corrosion and degradation (b). Moreover, significant detachment from the GC substrate can be observed (b). Contrarily, for the $Cu_{0.88}Ag_{0.12}$ tandem catalyst, the presence of Ag is found to improve the durability of the particles during eCO_2RR in CO_2-saturated $KHCO_3$ electrolyte (d).

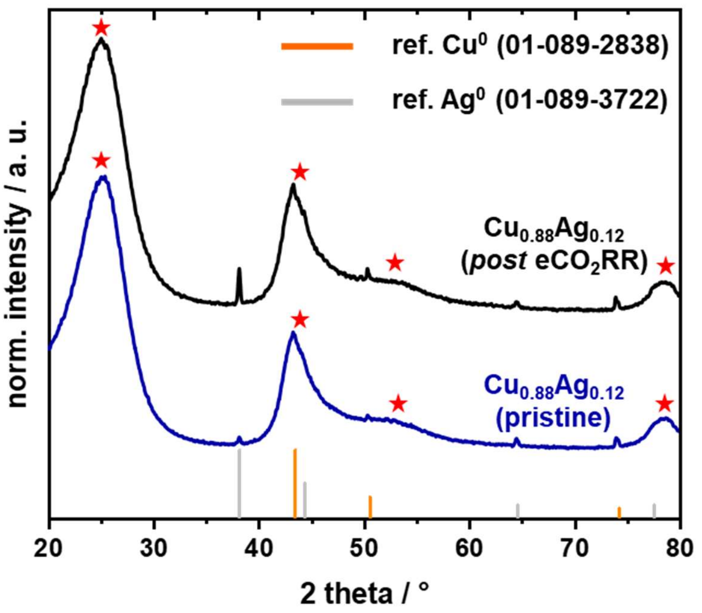

Supplementary Fig. 5 GI-XRD analyses of pristine $Cu_{0.88}Ag_{0.12}$ prior to (black) and after (blue) eCO_2RR. Both patterns reveal the presence of a metallic Cu^0 phase as the predominant phase in the bulk of the NPs. Additionally, reflections corresponding to metallic Ag^0 are indicated. The broad reflections indicated by asterisks (red) can be attributed to the GC substrate. No indications for any pronounced bulk oxidation of the tandem catalyst particles are found after the eCO_2RR test. The measurements were carried out at a grazing incidence angle of 0.3° for the incoming X-ray beam.

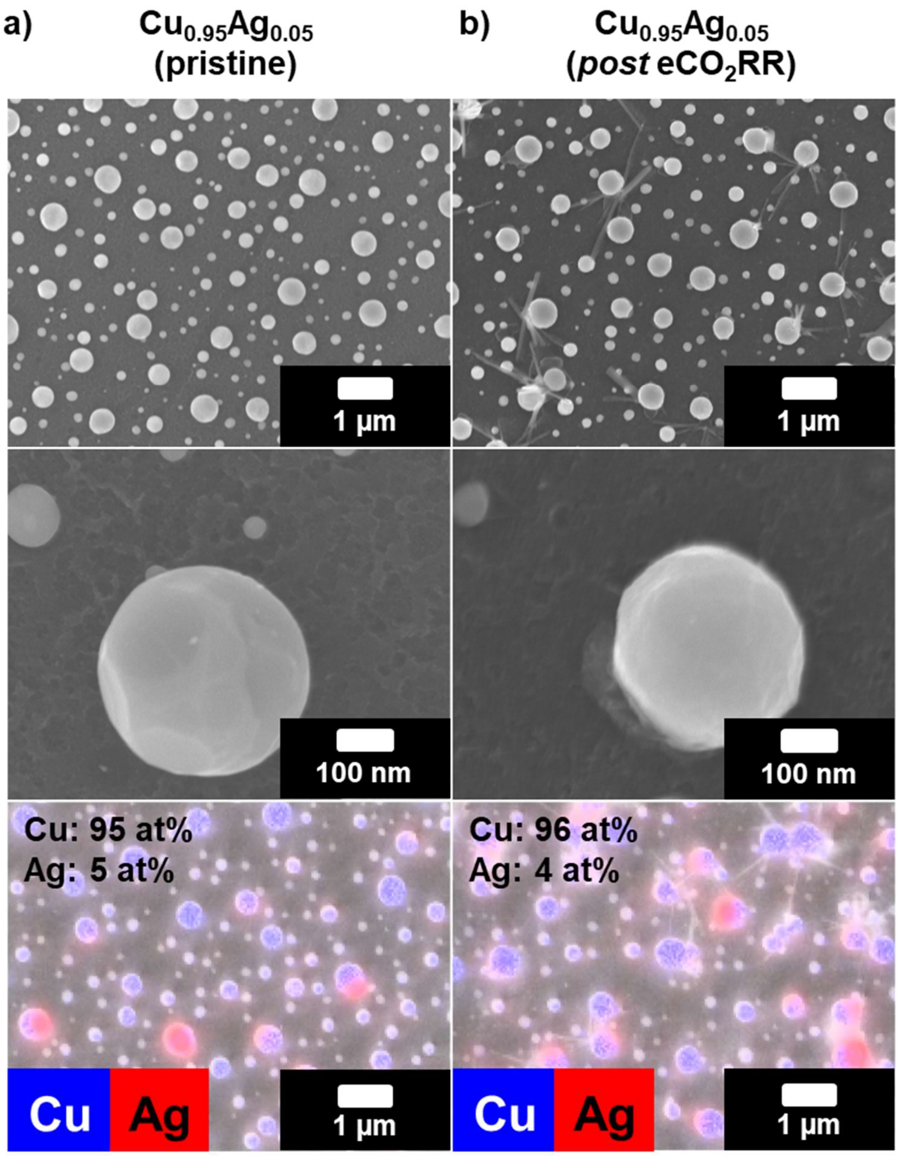

Supplementary Fig. 6 SEM images of $Cu_{0.95}Ag_{0.05}$ NPs prior to (a) and after (b) eCO_2RR. After the 6 h eCO_2RR test, no indications for pronounced corrosion, degradation or particle detachment from the GC substrate can be found. Similar to the $Cu_{0.88}Ag_{0.12}$ tandem catalyst, the presence of low amounts of Ag (~ 5 at%) drastically improves the durability of the particles during eCO_2RR in CO_2-saturated $KHCO_3$ electrolyte (b). SEM-EDX mappings (bottom row) reveal a slight loss of Ag in the NPs after the eCO_2RR test (b). Thus, it can be hypothesized that Ag species undergo leaching processes over time, leading to a reduced Ag/Cu ratio in the spent catalyst.

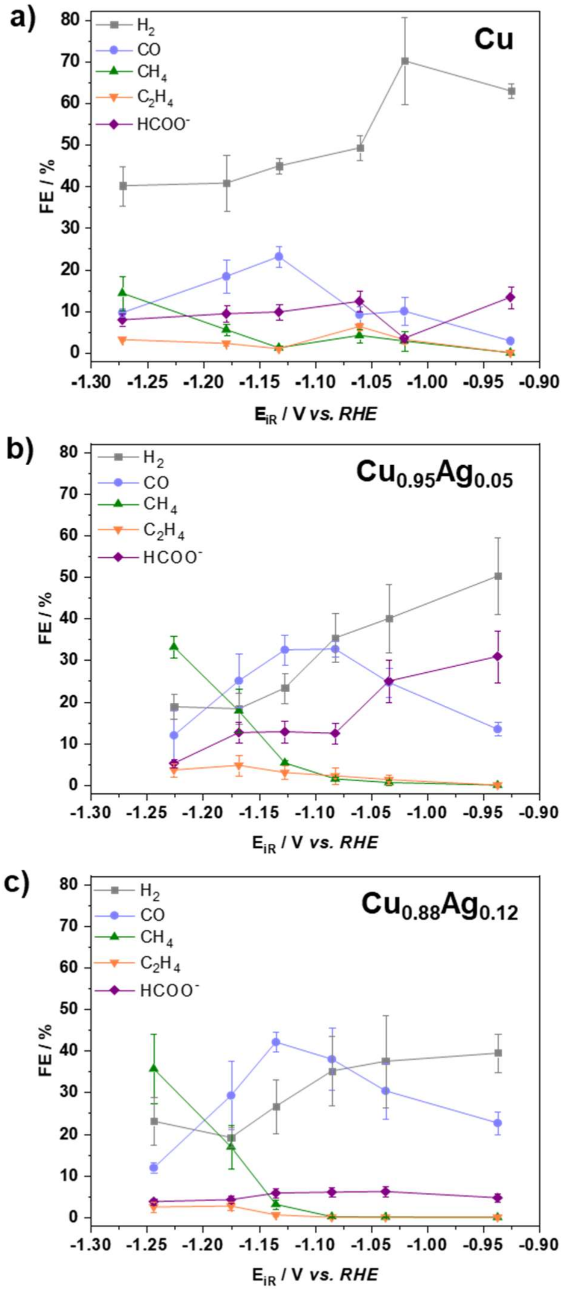

Supplementary Fig. 7 Potential-dependent Faradaic efficiencies for bare Cu (a), $Cu_{0.95}Ag_{0.05}$ (b) and $Cu_{0.88}Ag_{0.12}$ (c) tandem catalyst systems. According to the findings from eCO_2RR tests conducted in an H-cell at room temperature, the introduction of Ag enhances C_1 product selectivity, particularly of CO and CH_4. In the case of the carbon-supported, monometallic Cu NP-based electrode (a), the essentially fully reduced, i.e. metallic, character favors the competing catalytic evolution of H_2 (HER) over CO_2 reduction, as previously reported for defect-free, flat Cu^0 surfaces.[1, 2] Evidently, Ag species in the tandem catalysts (b, c) effectively suppress the competing HER over the entire investigated potential range. For $Cu_{0.95}Ag_{0.05}$, an increased $HCOO^-$ product selectivity is observed with respect to monometallic Cu and $Cu_{0.88}Ag_{0.12}$. Overall, a trend of decreasing $HCOO^-$ FEs for more cathodic potentials is found. Note that all potentials were corrected for Ohmic loss (*iR* drop; full compensation) and are stated *vs. RHE*. Beyond that, the corresponding error bars from at least three independent measurements are given (standard deviations).

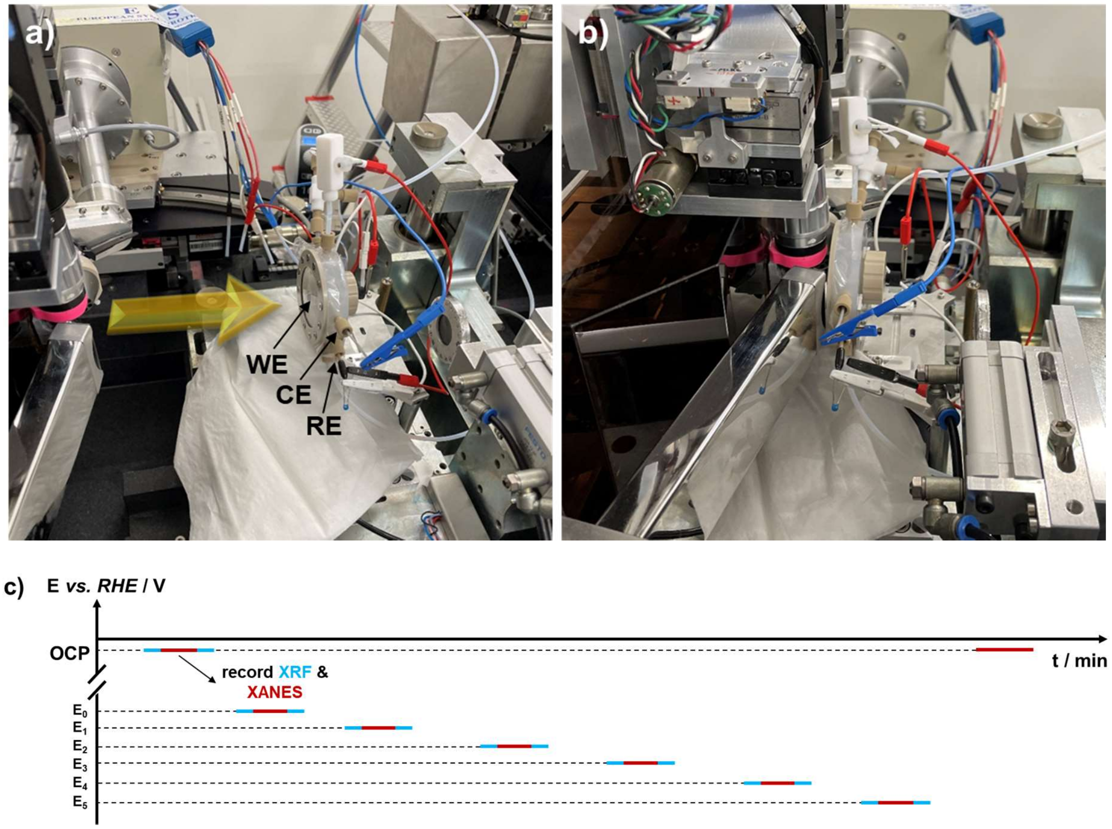

Supplementary Fig. 8 *In situ* nanofocused XAS setup. Representative photographs (a, b) show the *in situ* nano-XAS setup at ID16B (ESRF), including the electrochemical cell with its electrical connections for working (WE), counter (CE) as well as reference (RE) electrodes. CO_2-saturated 0.1 M $KHCO_3$ electrolyte is continuously pumped through the cell, which is sealed by a thin membrane. The WE is located in the center of the cell, on which the incoming beam (schematically illustrated as yellow arrow in (a)) is focused. In (b), the cell is located in the X-ray focal plane. The current response was recorded at varying potentials (chronoamperometry) during the acquisition of the XAS data (c). Prior to and after each XAS measurement at a certain potential, nanofocused XRF maps were acquired in order to obtain information about the position of each particle (light blue steps in (c)). Particularly, sample stability is critical and beam damage must be carefully controlled, e.g. by adjusting the electrolyte flow in order to prevent extensive local heating and radical formation close to the surface of the WE. In this context, an effective removal of evolving gas bubbles needs to be guaranteed in order to prevent particle detachment from the electrode or blocking of active sites.

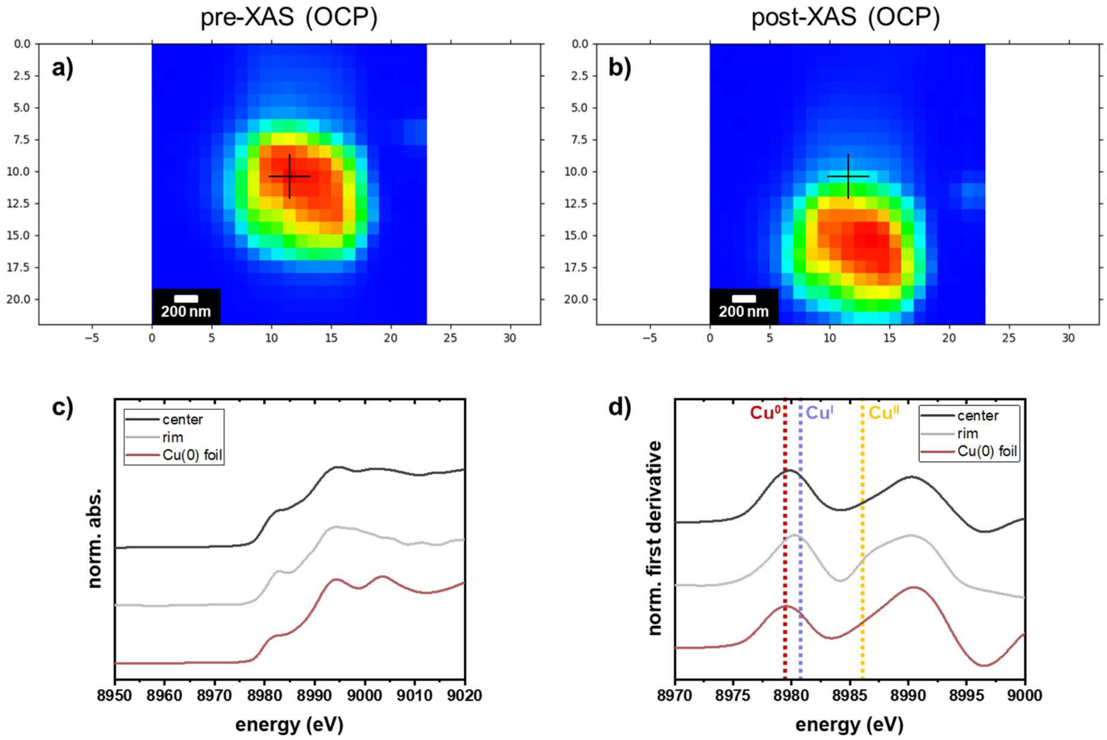

Supplementary Fig. 9 ***In situ*** **nanofocused XRF maps (Cu; a, b) and Cu K-edge XANES spectra (c, d) of an individual bimetallic NP ($Cu_{0.88}Ag_{0.12}$ catalyst) at OCP.** Derived from the Cu K X-ray emission signals, the obtained XRF maps (a, b) display the positions of a single $Cu_{0.88}Ag_{0.12}$ NP prior to (a) and after (b) the acquisition of the XANES spectra. A black cross (a, b) indicates the position at which nano-XAS data were collected (particle center: (a); particle rim: (b)). As a reference, a Cu K-edge XANES spectrum of a Cu^0 metal foil is given (c, d). At OCP, predominantly metallic Cu^0 species are present in the bulk of the tandem $Cu_{0.88}Ag_{0.12}$ NP. Toward the outer particle rim, oxidized $Cu^{I/II}$ species are indicated by the analysis of the location-specific Cu K-edge XANES spectra (c, d). Note that the position of the dotted vertical lines for Cu^I and Cu^{II} species (d) was calculated based on previously reported shifts between Cu^0 metal and a Cu_2O or a CuO reference, respectively.[3] Due to a strong overlap between the Ag L signal at 2.98 keV and the K lines of Ar (air) and K (electrolyte) at 2.96 and 3.31 keV, respectively, as well as a pronounced self-absorption of the Ag L lines emitted within the NPs by the Cu species, a reliable evaluation of the Ag L line maps is not feasible. Nevertheless, the findings from the analysis of multiple individual NPs *via* nano-XAS based on Cu maps unambiguously reveal the presence of metallic Cu^0 species throughout the entire bulk phase of an individual NP during eCO_2RR conditions. For further insights into the role of the Cu-Ag interface or the Ag domain, Ag K edge experiments would be necessary.

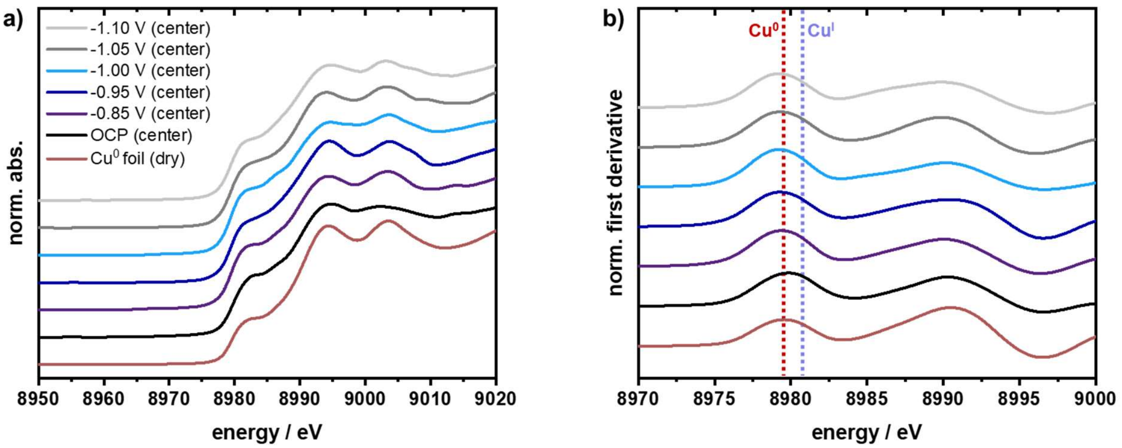

Supplementary Fig. 10 ***In situ*** **nanofocused Cu K-edge XANES spectra (a, b) at the center of individual $Cu_{0.88}Ag_{0.12}$ tandem catalyst NPs.** For each spectrum plotted above, XRF mappings were utilized in order to focus the X-ray beam to the center of individual particles. In general, the nanofocused spectra display well-defined XANES regions and less defined EXAFS regions, thereby impeding reliable analyses of the fine structures. Note that all potentials stated were converted to the *RHE* scale and *iR*-corrected. Briefly, for potentials more negative than about -0.85 V_{RHE}, metallic Cu^0 represent the main species in the bulk of the tandem catalyst NPs. The findings indicate a complete reduction of (partially) oxidized $Cu^{I/II}$ species in the center of a particle below an applied potential of -0.85 V_{RHE}. Here, it has to be mentioned that X-ray induced beam damage effects cannot be entirely ruled out. Yet, a reduced exposure time in the range of several seconds was maintained in order to reduce beam-induced variations on the oxidation states of the bulk Cu species.

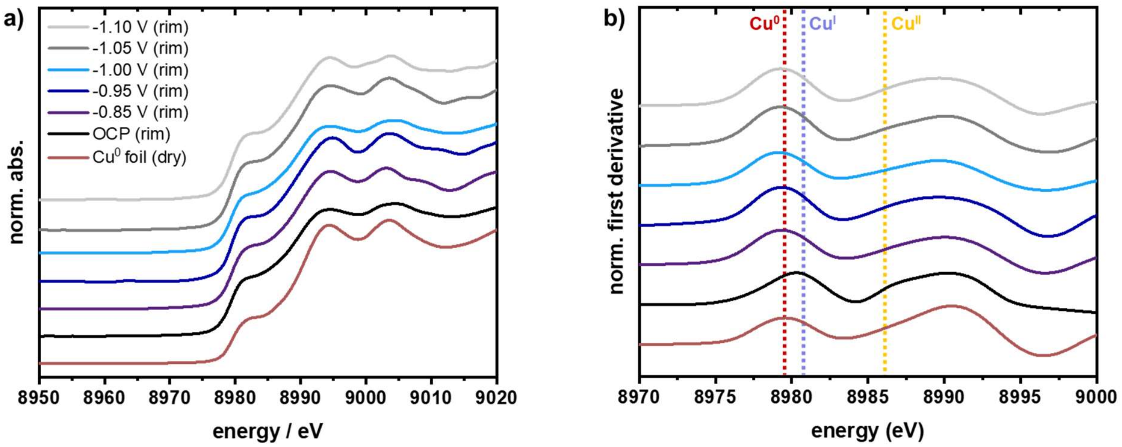

Supplementary Fig. 11 *In situ* nanofocused Cu K-edge XANES spectra (a, b) at the outer particle rim of isolated $Cu_{0.88}Ag_{0.12}$ tandem catalyst NPs. For each spectrum plotted above, XRF mappings were utilized in order to focus the X-rays to the outer rim of individual particles, i.e. close to the interface to the electrolyte. Albeit XAS, in general, represents a bulk-sensitive method, nanofocused XANES can provide insights into the chemical structure (oxidation states) close to a particle's surface. Note that all potentials stated were converted to the *RHE* scale and *iR*-corrected. At OCP, a significant amount of (partially) oxidized $Cu^{I/II}$ species is indicated (b), which can be attributed to the thermodynamically favored oxidation of Cu atoms by the exposure to air and humidity after the synthesis of the electrodes. Importantly, negative potentials of -0.85 V_{RHE} (*iR*-compensated) already lead to a complete reduction to Cu^0 – similar to the observations mentioned above for the analyses of a particle's center. Compared to the latter, lower signal-to-noise ratios were found due to reduced absorption cross-sections close to the particle-electrolyte interface, potentially leading to a higher degree of variation in each spectral shape. Apart from slight variations in the electronic structure, dynamic geometric rearrangements/restructuring close to the particle's surface may occur, ultimately affecting spectral shapes. As noted above (Supplementary Fig. 10), beam damage (e.g. local heating) cannot be entirely ruled out. Visual inspection of the NPs on the spent electrodes for the *in situ* characterization, however, did not provide any evidence for pronounced particle growth, aggregation or corrosion.

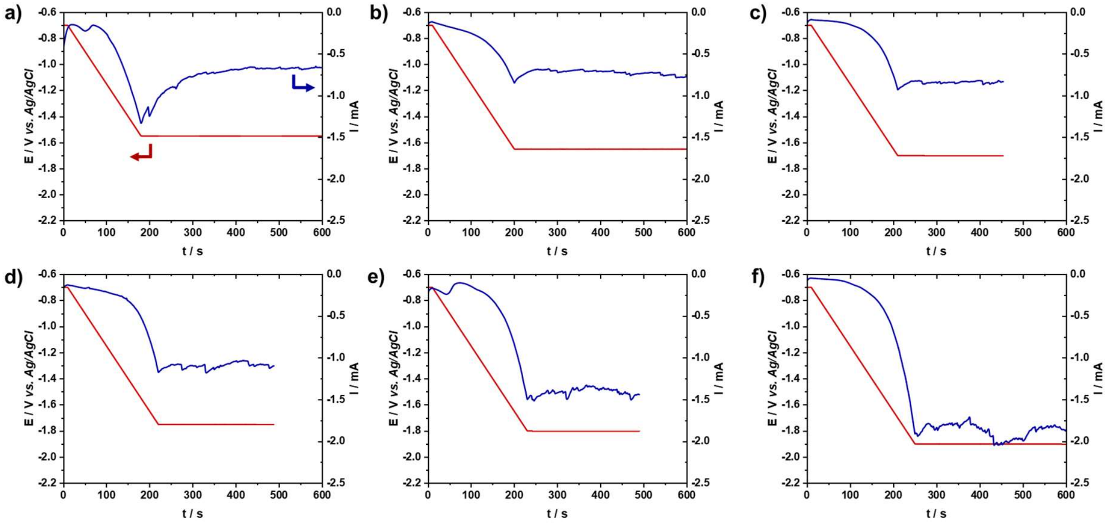

Supplementary Fig. 12 Representative chronoamperometry plots (a - f) of $Cu_{0.88}Ag_{0.12}$ tandem catalyst NPs during *in situ* nano-XAS at ID16B. Red and blue graphs correspond to the applied potential (*vs. Ag/AgCl*) and the current response, respectively, during the *in situ* nano-XAS experiments using CO_2-saturated 0.1 M $KHCO_3$ electrolyte at room temperature. Note that the current signal has not been corrected for Ohmic loss (*iR* drop) inside the cell. In addition, at least three different particles were investigated at each potential step. Therefore, repetitive chronoamperometry experiments were conducted during the acquisition of the nano-XAS data. We hypothesize that drops in current at a certain potential can either be related to the formation of gas bubbles - blocking active catalytic sites - or to the loss of active catalyst material, e. g. as a result of (beam-induced) particle detachment.

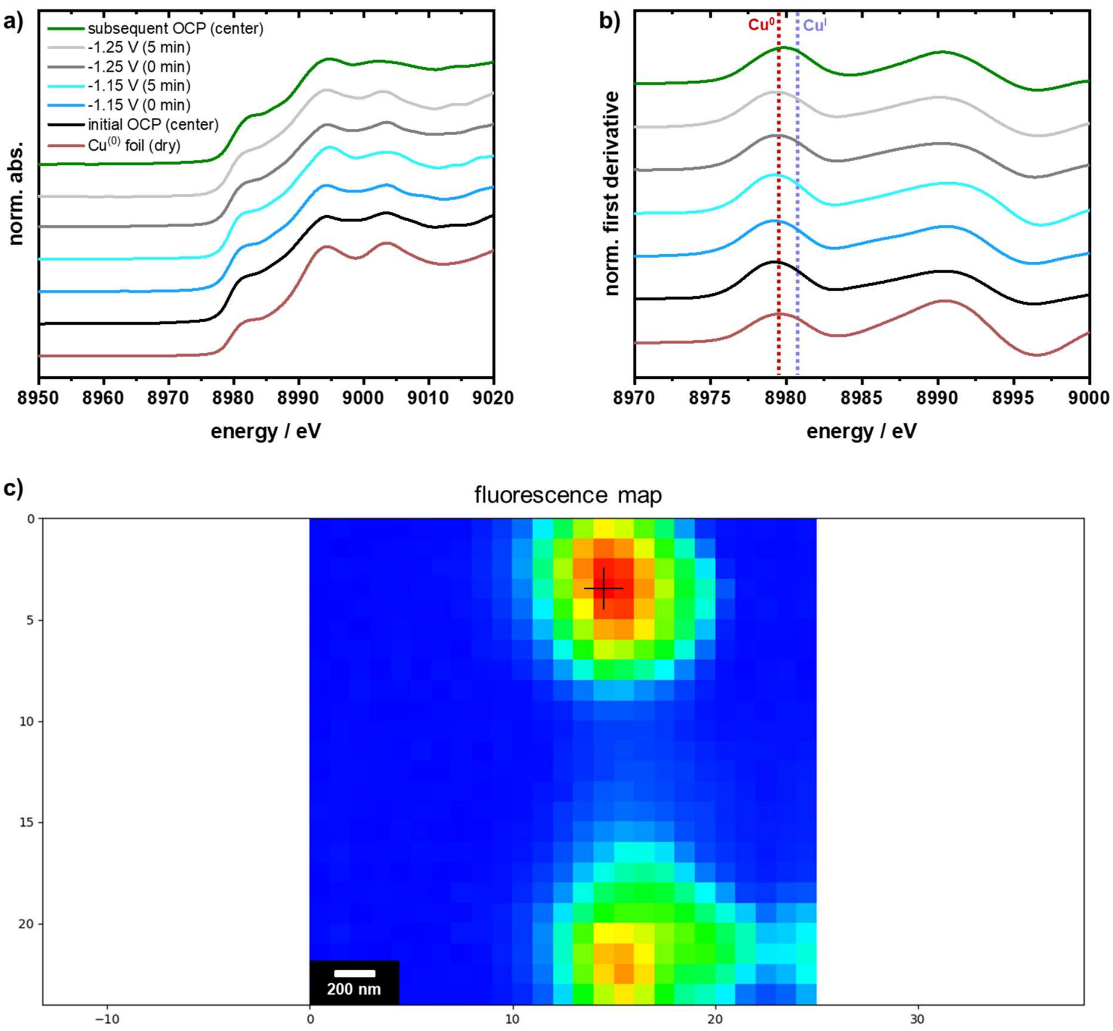

Supplementary Fig. 13 Investigation of structural dynamics of an individual $Cu_{0.88}Ag_{0.12}$ NP during eCO_2RR *via* nano-XAS at ID16B. For each spectrum plotted in (a) & (b), consecutive XRF mappings were utilized in order to focus the X-rays to the center of the same individual particle (c). Note that all potentials stated were converted to the *RHE* scale and *iR*-corrected. According to the XANES spectra shown in (a) & (b), metallic Cu^0 remain the predominant species after approx. 5 min at negative potentials of -1.15 and -1.25 V_{RHE}. Analysis of the acquired spectra after switching back to OCP (green traces in a, b) does not reveal clear evidence of pronounced re-oxidation of the bulk Cu species. Note that prolonged *in situ* measurement durations were hampered by extensive gas bubble formation at the membrane/WE interface.

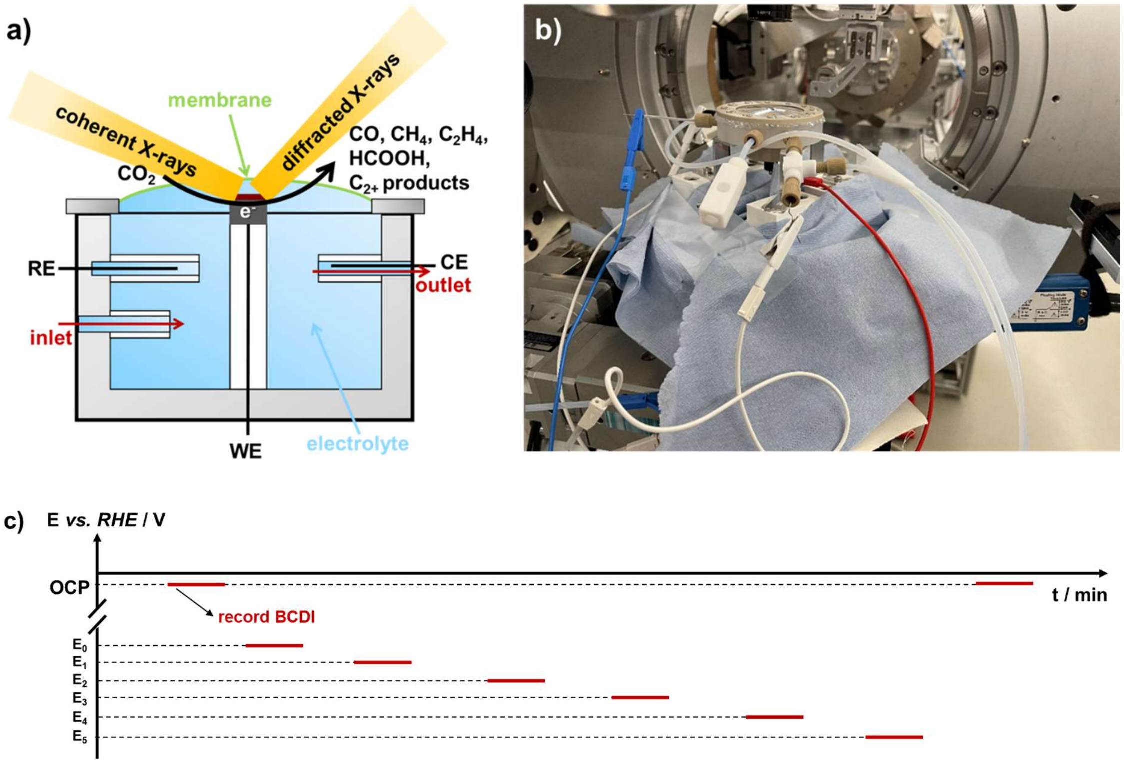

Supplementary Fig. 14 ***In situ*** **nanofocused BCDI setup (a, b) and measurement protocol (c) for the identification of three-dimensional strain in Cu-Ag model catalyst systems during eCO_2RR.** A schematic illustration (a) displays the geometry of the customized *in situ* cell and the incoming coherent X-rays. A representative photograph (b) shows the *in situ* nano-BCDI setup at P10 (PETRA synchrotron, DESY), including the electrochemical cell and its electrical connections for WE, CE and RE. CO_2-saturated 0.1 M $KHCO_3$ electrolyte is continuously pumped through the cell, which is sealed by a thin membrane. The WE is located at the center of the cell, on which the incoming beam is focused. The current response was recorded at varying potentials (chronoamperometry) during the acquisition of the BCDI data (c). In principle, lattice displacements result in a shift of the path length (i.e. a phase shift) of the scattered X-rays. Ideally, fundamental insights into structural information, such as defects, exposed facets or the particle shape can be obtained under eCO_2RR working conditions (*cf.* Fig. 2j, MAIN). Therefore, several challenges need to be overcome, such as a sufficient stability of the particles against pronounced radiation damage as well as against undesirable translational or rotational movements under illumination. The same *in situ* cell was used for both nano-XAS and -BCDI. However, for the diffraction experiments, a horizontal cell alignment is required (*cf.* Fig. 4a, MAIN).

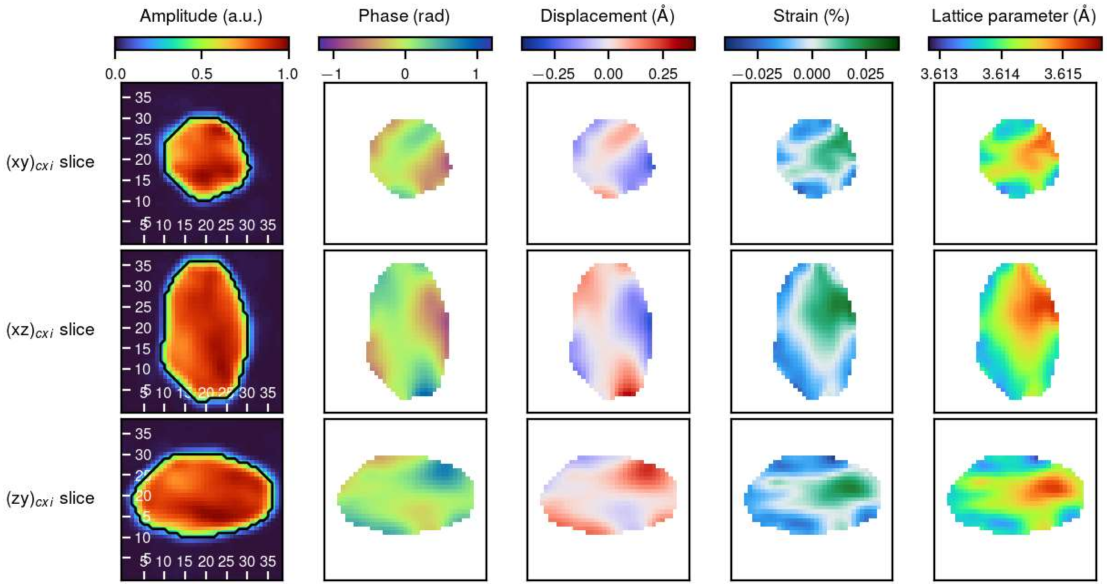

Supplementary Fig. 15 ***In situ*** **nano-BCDI evaluation of an individual NP of a $Cu_{0.95}Ag_{0.05}$-based electrode at OCP.** Overall, sample stability for $Cu_{0.95}Ag_{0.05}$ was lower than for $Cu_{0.88}Ag_{0.12}$ NP-based electrodes, thwarting further reconstruction under eCO_2RR conditions. Here, a representative reconstruction of an individual NP at OCP is shown, revealing the presence of both compressive (blue) and tensile (green) strain. From the calculated average d-spacing (approx. 2.087 Å), an averaged lattice constant of 3.614 Å can be derived, indicating a [111]-oriented Cu crystal featuring minute amounts of Ag, if any. The NP is oriented in the CXI [https://www.cxidb.org/cxi.html] coordinate system. Slices of the reconstructed amplitude, phase, displacement, strain and lattice parameter are shown. The phase, displacement and strain are measured along the [111]-direction.

Supplementary References

1. Grosse P, Gao D, Scholten F, Sinev I, Mistry H, Roldan Cuenya B. Dynamic Changes in the Structure, Chemical State and Catalytic Selectivity of Cu Nanocubes during CO2 Electroreduction: Size and Support Effects. *Angewandte Chemie International Edition* **57**, 6192-6197 (2018).

2. Scholten F, Nguyen K-LC, Bruce JP, Heyde M, Roldan Cuenya B. Identifying Structure–Selectivity Correlations in the Electrochemical Reduction of CO2: A Comparison of Well-Ordered Atomically Clean and Chemically Etched Copper Single-Crystal Surfaces. *Angewandte Chemie International Edition* **60**, 19169-19175 (2021).

3. Lin S-C*, et al.* Operando time-resolved X-ray absorption spectroscopy reveals the chemical nature enabling highly selective CO2 reduction. *Nature Communications* **11**, 3525 (2020).
